# Nanotechnology and Artificial Intelligence in Dyslipidemia Management—Cardiovascular Disease: Advances, Challenges, and Future Perspectives

**DOI:** 10.3390/jcm14030887

**Published:** 2025-01-29

**Authors:** Ewelina Młynarska, Kinga Bojdo, Hanna Frankenstein, Natalia Kustosik, Weronika Mstowska, Aleksandra Przybylak, Jacek Rysz, Beata Franczyk

**Affiliations:** 1 Department of Nephrocardiology, Medical University of Lodz, 90-549 Łódź, Poland; 2 Department of Nephrology, Hypertension and Internal Medicine, Medical University of Lodz, 90-549 Łodz, Poland

**Keywords:** dyslipidemia, cardiovascular disease, emerging lipid-lowering therapies, nanotechnology, artificial intelligence, cardiovascular risk-related biomarkers

## Abstract

This narrative review explores emerging technologies in dyslipidemia management, focusing on nanotechnology and artificial intelligence (AI). It examines the current treatment recommendations and contrasts them with the future prospects enabled by these innovations. Nanotechnology shows significant potential in enhancing drug delivery systems, enabling more targeted and efficient lipid-lowering therapies. In parallel, AI offers advancements in diagnostics, cardiovascular risk prediction, and personalized treatment strategies. AI-based decision support systems and machine learning algorithms are particularly promising for analyzing large datasets and delivering evidence-based recommendations. Together, these technologies hold the potential to revolutionize dyslipidemia management, improving outcomes and optimizing patient care. In addition, this review covers key topics such as cardiovascular disease biomarkers and risk factors, providing insights into the current methods for assessing cardiovascular risk. It also discusses the current understanding of dyslipidemia, including pathophysiology and clinical management. Together, these insights and technologies hold the potential to revolutionize dyslipidemia management, improving outcomes and optimizing patient care.

## 1. Methods Section

This review aims to evaluate current therapeutic strategies for dyslipidemia, focusing on novel approaches and their clinical applications. Evidence was synthesized from the peer-reviewed literature published before January 2025. A systematic search was conducted using the PubMed, Scopus, Google Scholar, and Web of Science databases. The search terms included “dyslipidemia”, “cardiovascular disease”, “lipid-lowering therapies”, “nanotechnology”, and “artificial intelligence”. The searches were limited to English-language articles. Additional articles were identified through the manual searches of the reference lists from the included studies.

The inclusion criteria focused on peer-reviewed studies, including narrative reviews, systematic reviews, meta-analyses, and clinical trials. Studies were excluded if they were not peer-reviewed (such as editorials or commentaries), focused on topics unrelated to the scope of this review, or were published in languages other than English.

## 2. Cardiovascular Disease—Risk Factors, Biomarkers, and Artificial Intelligence Correlation

Cardiovascular disease (CVD) is the leading group of non-communicable diseases globally, responsible for around one-third of all deaths worldwide [1]. CVD is a general term for heterogeneous conditions affecting the heart or blood vessels, mainly such as coronary artery disease (CAD) and atherosclerosis [2,3]. Inflammatory activation and endothelial dysfunction play central roles in the onset and progression of atherosclerosis, contributing to an increased risk of cardiovascular events like myocardial infarction [4]. There are several risk factors that contribute to the development of CVD like age, gender (men over 45 and women over 55, particularly after menopause), genetics factors, smoking, lack of physical activity, high blood pressure, obesity, high cholesterol and triglyceride levels, and diabetes mellitus (DM) [5]. As we know, there is a high correlation between lipid disorders and the development of CVD. An increased level of circulating apo-B-containing lipoproteins has long-been identified as a central causal risk factor for coronary heart disease [2].

We can also distinguish biomarkers that correlate with CVD. Traditional biomarkers include high-sensitivity C-reactive protein and the cardiac troponins I or T (cTns). These biomarkers, especially cTns, are released into the bloodstream when cardiac myocytes undergo necrosis. Testing for these biomarkers is critical in diagnosing, stratifying risk, and managing patients with cardiovascular disease [6]. In the seminal JUPITER trial, Ridker et al. demonstrated that healthy community-dwellers with low concentrations of low-density lipoprotein (LDL) cholesterol, but increased concentrations of CRP (>2 mg/L), strongly benefited from statin therapy, with a reduced incidence of cardiovascular events [7,8]. While cTns are considered the gold standard for diagnosing acute CVD caused by cardiomyocyte necrosis, false-positive results can present challenges [9]. Elevated cTn levels are not limited to ischemic injuries but are also observed in nonischemic myocardial damage, such as myocarditis and cardiotoxicity, as well as in other conditions involving multifactorial injuries, including congestive heart failure and pulmonary embolism [7,8]. In 2009, two landmark studies in The New England Journal of Medicine highlighted the superior diagnostic performance of high-sensitivity troponin assays compared to conventional assays, particularly in early presenters, enabling faster acute myocardial infarction (AMI) confirmation. By 2015, a single high-sensitivity cTnI measurement below 5 ng/L was validated as a reliable cutoff for ruling out AMI in low-risk emergency patients, with a 99.6% negative predictive value. These findings were consistent across age and sex subgroups and confirmed in multiple cohorts. However, diagnostic performance was less robust for patients presenting within two hours of symptom onset [9].

Creatine Kinase-MB (CK-MB) is an enzyme predominantly found in heart muscle cells, and its presence in the blood serves as a biomarker for myocardial damage, such as that caused by a heart attack. Rapid fluctuation makes CK-MB particularly useful for identifying reinfarction, or a second heart attack that occurs shortly after an initial one. However, CK-MB is less specific than cardiac troponins for detecting myocardial injury [10].

Heart-type fatty acid-binding protein (H-FABP) is a small protein rapidly released into the bloodstream within 1–3 h of myocardial injury, making it valuable for the early diagnosis of AMI. While less specific than troponins due to potential elevation in skeletal muscle injury and renal impairment, H-FABP complements troponins by enhancing early sensitivity in diagnosing AMI. Elevated H-FABP levels are also linked to worse outcomes in acute coronary syndrome (ACS) and heart failure (HF), offering both diagnostic and prognostic insights [10,11].

Another commonly used biomarker is the N-terminal prohormone of brain natriuretic peptide (NT-proBNP) that correlates to myocardial stretch. Elevated levels of NT-proBNP in a patient’s blood upon arrival at the emergency room are strongly linked to an increased likelihood of a heart failure diagnosis. Additionally, higher NT-proBNP levels at the time of hospital admission are correlated with a higher risk of in-hospital mortality [7]. Despite the high predictive value of NT-proBNP, the GUIDE-IT study, a large randomized controlled trial evaluating NT-proBNP-guided therapy for heart failure with reduced ejection fraction, was terminated early due to futility, showing no outcome differences compared to the standard care and current evidence does not support NT-proBNP-guided therapy for chronic heart failure [9].

Copeptin, a precursor of arginine vasopressin, is another biomarker linked to both ischemic events and heart failure. The vasopressin system is activated after acute myocardial infarction, so copeptin levels rise shortly after an ischemic episode and are associated with an increased risk of mortality and the development of new-onset heart failure, especially in those with an elevated NTproBNP [12,13].

The biomarkers described above are classified as classical biomarkers. However, with the advancement of technology, new biomarkers are emerging, such as microRNAs (mi-RNAs). Mi-RNAs are small, non-coding RNA molecules that play an essential role in regulating gene expression. They have distinct expression patterns and release during different stages of CVDs. For AMI, miR-29b has been identified as an important biomarker, while miR-1, miR-133, and miR-328 are relevant for AMI, arrhythmias, and HF. For HF, miRNAs like miR-18, miR-37, miR-126, miR-210, miR-221, and miR-1254 have diagnostic and prognostic significance. Additionally, miR-21, miR-208, and miR-499 serve as biomarkers for both AMI and HF, enhancing the understanding of these conditions. Analyzing miRNA profiles provides deeper insights into the molecular mechanisms behind CVDs and can reveal potential targets for therapy and tools for diagnosis [7,10].

Traditionally, statistical models incorporating risk factors and biomarkers have been widely used to predict and assess the progression of CVD. However, with technological advancements, the use of AI is rapidly gaining popularity as a method for evaluating patient risk and predicting CVD outcomes. Most studies on AI in CVD have been carried out in developed countries. These studies cover various applications, including the use of machine learning (ML) for the risk stratification of CVD, employing natural language processing to extract data from clinical and pathological reports, integrating AI into clinical decision support systems, and applying AI for the prognosis and treatment of CVD [14]. One notable example is the use of ML in risk stratification, which has demonstrated improvements in identifying high-risk patients and reducing unnecessary treatments for low-risk individuals presented by Kakadiaris et al. (2018). The ML-based calculator developed in their study surpassed the traditional ACC/AHA Risk Calculator by identifying 13% more high-risk patients and reducing therapy for low-risk individuals by 25% [15]. AI-based polygenic risk score (PRS) modeling approaches have significant potential to improve personalized CVD treatments. By integrating multi-omics data (genomic, transcriptomic, proteomic, and metabolomic), these methods provide a deeper understanding of molecular pathways and enhance the accuracy of PRS models. AI techniques are particularly useful for identifying complex patterns in high-dimensional genetic data, enabling more precise connections between genetic variations and disease risk. Moreover, AI algorithms can continually learn from new data, improving their performance and offering more accurate risk assessments over time [16].

## 3. Dyslipidemia—Current Understanding

Dyslipidemia refers to an abnormality in the normal levels of one or more lipid components in the blood, such as triglycerides (TGs), total cholesterol (TC), high-density lipoprotein cholesterol (HDL-C), and low-density lipoprotein cholesterol (LDL-C) [17]. Cholesterol and triglycerides are water-insoluble and require transport with proteins such as lipoproteins. These complex particles consist of a core of cholesterol esters and triglycerides surrounded by free cholesterol, phospholipids, and apolipoproteins, which play key roles in lipoprotein structure, receptor binding, assembly, and enzyme regulation. Plasma lipoproteins are classified into seven types based on size, composition, and apolipoproteins: chylomicrons, chylomicron remnants, very low-density lipoproteins (VLDLs), VLDL remnants (IDL), LDL, HDL, and lipoprotein (a) (Lp(a)). Chylomicron remnants, VLDL, IDL, LDL, and Lp(a) promote atherosclerosis, while HDL has a protective role [18]. Dyslipidemia can arise from three potential causes: intrinsic, extrinsic, or a combination of genetic and environmental factors. Secondary dyslipidemias are associated with risk factors linked to other medical conditions or environmental influences, whereas primary dyslipidemias represent a varied group of disorders stemming from genetic causes, either monogenic or polygenic in nature [17]. As we know, dyslipidemia shows a high correlation with obesity and CVD risk. In individuals with adiposity, a commonly observed lipid profile is adiposopathic dyslipidemia (also referred to as “atherogenic dyslipidemia”). This pattern is characterized by elevated blood triglyceride levels, reduced HDL-C levels, increased non-HDL-C levels, elevated apolipoprotein B levels, a higher number of LDL particles, and an increase in small dense LDL particles [19]. Lipoproteins, such as chylomicrons and VLDL, play a crucial role in lipid metabolism, transporting triglycerides from the intestine and liver to tissues like adipose tissue, muscles, and the heart. Lipoprotein lipase (LPL) breaks down triglycerides, releasing free fatty acids. As triglycerides are depleted, these lipoproteins are converted into remnant lipoproteins, which are absorbed by the liver or further processed into LDL. The accumulation of LDL and remnant lipoproteins in the arterial walls contributes to atherosclerosis, a lipid-driven process that leads to atherosclerotic cardiovascular disease (ASCVD) [20,21,22,23]. Atherosclerotic plaque formation depends on LDL-C and ApoB-containing lipoproteins, and prolonged exposure to these particles increases risk [24]. Lp(a) and inflammation are linked to ASCVD risk even in statin-treated individuals [25]. Complex classifications analyzing the factors correlated with the development of dyslipidemia could more effectively and efficiently identify potential patients. Therefore, the integration of artificial intelligence in assessing risk, diagnosis, and complications is crucial.

## 4. Current Recommendations in Lipid-Lowering Therapies

Dyslipidemia is a major factor in atherosclerosis and CVD. The public health priority is to achieve optimal lipoprotein levels. In general, treatment starts with lifestyle changes, including a healthy diet (low in sodium, saturated fats, and alcohol) and regular physical activity instead of sedentary life. In addition to these modifications, pharmacological treatment may be necessary [26,27].

It should be emphasized that managing weight is also essential for preventing and reducing cardiovascular risks, especially for people with obesity whose body mass index (BMI) is 30 kg/m^2^ or higher or those with a BMI between 25 and 29.9 kg/m^2^, classified as overweight, who have two or more additional risk factors [27]. For individuals with extreme obesity or those with comorbidities, bariatric surgery and medications like orlistat, naltrexone, bupropion, or high-dose liraglutide have demonstrated significant effectiveness [28].

The Mediterranean Diet emphasizes the consumption of fruits, vegetables, whole grains, legumes, nuts, and olive oil, with a moderate intake of fish and red wine, offering significant heart health benefits [29]. Similarly, the Dietary Approaches to Stop Hypertension (DASH) diet, which focuses on fruits, vegetables, and low-fat dairy, effectively lowers blood pressure and cardiovascular risk, with a 20% reduction in disease risk linked to adherence [28,30].

It is also important to consume healthy fats, as they play a crucial role in maintaining optimal lipid profiles and supporting overall cardiovascular health. Fats are classified by origin and structure, with animal fats being rich in saturated fatty acids (SAFAs) and plant fats containing more unsaturated fatty acids like monounsaturated (MUFAs) and polyunsaturated (PUFAs) fats [31]. Omega-3 and omega-6 PUFAs, particularly Eicosapentaenoic acid (EPA) and Docosahexaenoic acid (DHA), help reduce triglycerides by 10–50% and have cardioprotective effects [32,33,34], with a recommended intake of 2–4 g/day for triglyceride reduction [32]. In contrast, trans fatty acids (TFAs) found in full-fat dairy and processed foods raise LDL cholesterol and lower HDL levels, worsening lipid profiles [32]. Phytosterols, plant compounds, can reduce LDL cholesterol by 6–12% at 2 g/day by decreasing cholesterol absorption [31].

### 4.1. Pharmacotherapy

#### 4.1.1. Statins

Statins were introduced to the pharmaceutical market in 1987, starting with lovastatin. Currently, they are the leading treatment for managing high cholesterol and preventing cardiovascular diseases. Over time, six statins have become available: two semi-synthetic statins (simvastatin and pravastatin) and four synthetic statins (fluvastatin, atorvastatin, rosuvastatin, and pitavastatin) [34]. The primary mechanism of this medicine involves inhibiting the enzyme 3-hydroxy-3-methyl-glutaryl-coenzyme A reductase (HMG-CoA). By limiting this enzyme’s activity, statins reduce cholesterol synthesis within liver cells, leading to lower intrahepatic cholesterol levels. This triggers an upregulation of LDL receptors (LDLRs) in liver cells, which helps remove LDL cholesterol from the bloodstream. This reduction in LDL-C by 1 mmol/L (38.6 mg/dL) has been shown to decrease the risk of cardiovascular events even by 21% [35]. High-intensity statin therapy, such as atorvastatin (40/80 mg) or rosuvastatin (20/40 mg per day), is recommended as the initial treatment to prevent cardiovascular events. In addition to lowering LDL-C, statins are also effective in reducing triglycerides in patients with hypertriglyceridemia. As with each treatment, statins can also cause side effects, which might limit their use in some patients. The most common aftermath is myalgia, affecting 1–10% of users. Other issues like myopathy, rhabdomyolysis, liver and kidney dysfunction, type 2 diabetes, and eye conditions like cataracts have also been reported. Rosuvastatin, in particular, has been linked to a slightly higher risk of new-onset diabetes compared to atorvastatin. When statin intolerance or nocebo effects occur, patients should be prescribed the highest tolerated dose as a baseline therapy [36].

#### 4.1.2. Fibrates

Fibrates are mainly administered to TG levels, and in some cases, to heighten HDL-C levels. They are capable of reducing TG levels by 25–50% and increasing HDL-C by 5–20%. Nevertheless, their effect on LDL-C differs depending on the patient’s TG levels. For patients with very high TG levels (>500 mg/dL), fibrates may paradoxically cause an increase in LDL-C. On the other hand, in cases where TG levels are not significantly high, fibrates can decrease LDL-C by 10–30%. Although they are occasionally used to raise HDL-C levels, their main therapeutic function is to lower TG levels. For patients with extremely high TG levels, those over 500 mg/dL, fibrates are often used to lower TG and prevent complications like pancreatitis. In these situations, fibrates can be combined with omega-3 fatty acids (fish oil) to achieve better lipid management [29].

#### 4.1.3. Niacin

Niacin, the first drug approved for dyslipidemia, is effective in severe hypertriglyceridemia (TG > 500–1000 mg/dL). It reduces total cholesterol and triglycerides by 20–50%, LDL-C, and Lp(a). It also increases HDL-C and promotes the transition to larger, less dense LDL particles. Unfortunately, niacin may increase the risk of progression to diabetes in obese people at risk of the disease. In such cases, niacin should be used with caution [29,37,38].

#### 4.1.4. PCSK9 Inhibitors

Proprotein convertase subtilisin/kexin type 9 inhibitors (PCSK9is) are a useful therapeutic replacement, especially for patients who cannot tolerate statins or require additional LDL-C reduction. These monoclonal antibodies, such as alirocumab and evolocumab, target PCSK9, a protein that degrades LDL receptors in hepatocytes. By repressing PCSK9, these drugs preserve LDL receptors, enhancing their ability to clear LDL-C from the blood. PCSK9is are administered subcutaneously every 2–4 weeks. They fundamentally lower LDL-C, non-HDL cholesterol, apoB, and lipoprotein A levels. Moreover, they provide cardiovascular benefits like reducing inflammation and stabilizing plaques. While generally well tolerated, with mild side effects like injection-site reactions and nasopharyngitis, their high cost remains a significant barrier to widespread use in clinical practice. Frequently joined with ezetimibe, they are an effective statin alternative [26,29,38,39].

#### 4.1.5. Inclisiran

Inclisiran is a synthetic small interfering RNA (siRNA) designed to inhibit PCSK9 production by binding to its mRNA. This mechanism enhances LDL receptor activity, leading to a significant reduction in LDL-C levels. Clinical trials, such as ORION-10 and ORION-11, assessed their effectiveness and safety in patients with CAD or other cardiovascular risk factors and elevated LDL-C levels (≥70 or ≥100 mg/dL, respectively). The studies showed that inclisiran lowered LDL-C levels by 50% compared to the placebo, with only mild and temporary injection-site reactions as side effects [40,41]. Inclisiran is given subcutaneously in a 300 mg dose. The first injection is followed by a second dose three months later. Subsequent doses are given every six months [34]. In addition to lowering LDL-C by 50–55%, inclisiran is well tolerated and offers a promising option for long-term cholesterol management in high-risk patients.

#### 4.1.6. Bempedoic Acid

Bempedoic acid is an oral adenosine triphosphate-citrate lyase (ACL) inhibitor that reduces LDL-C levels by decreasing hepatic cholesterol synthesis. It is mainly prescribed in combination with ezetimibe for patients who are intolerant to statins. Bempedoic acid is safe. Moreover, it reduces the risk of reinfarction [26,29,38].

#### 4.1.7. Ezetimibe

Ezetimibe inhibits cholesterol absorption in the intestines and lowers LDL-C by approximately 20%. The drug’s effectiveness depends on the presence of the Niemann-Pick1-like protein. By decreasing cholesterol delivery to the liver, it enhances the liver’s LDL receptors. Ezetimibe is commonly used as an adjuvant medication therapy when statin treatment alone is insufficient or in patients who cannot tolerate statins. Additionally, it has the added benefit of improving glycemic control in diabetic patients [28,36,37]. Table 1 provides a detailed comparison of lipid-lowering medications, summarizing the information discussed in Section 4. 

## 5. Nanotechnology in Lipid-Lowering Therapies

### 5.1. Negatively Charged Liposome Nanoparticles

The understanding and application of nanoscience through nanotechnology began to expand rapidly in the early 2000s, sparking a transformative revolution that impacted nearly every field of science. Nanoscience focuses on structures ranging in size from 1 to 100 nanometers, while nanotechnology converts this knowledge into practical applications [44]. They have revolutionized the field of medicine, significantly enhancing the precision and effectiveness of treatments. This progress is primarily driven by nanoparticles (NPs), which enable targeted drug delivery, improve bioavailability, and reduce side effects by interacting with biological systems at the molecular and cellular levels [45]. These nanoscale carriers—including liposomes, dendrimers, and polymeric nanoparticles—allow for the controlled release and precise localization of therapeutic agents, revolutionizing disease treatments such as cancer, cardiovascular conditions, and infections.

This field continues to grow, providing innovative solutions to medical challenges. Meaningful and promising advancements are currently being made in anticancer therapy [46], infectious diseases, ophthalmology [47], and hypercholesterolemia which is the primary focus of our narrative review.

The earliest drug NPs were biomimetic, mimicking the micelles and liposomes naturally present in the body [48]. Liposomes are spherical vesicles composed primarily of phospholipids—amphiphilic molecules that contain both hydrophilic (water-attracting) heads and hydrophobic (water-repelling) tails. “The ideal NP carriers should be biodegradable, stable, non-immunogenic, easy to fabricate, cost-effective, and able to release their payloads only at the target site” [48]. Liposomes, which are mainly composed of phospholipids, are biocompatible, biodegradable, and non-immunogenic, which make them ideal carriers. They can encapsulate almost any drug or complex molecule, regardless of its hydrophobic or hydrophilic nature [49].

An effective liposomal formulation, incorporating the previously mentioned characteristics, can be achieved by selecting an optimal lipid composition, applying tailored functionalization, and designing a targeted delivery strategy. The parameters include stability depending on the surface charge, lipid rigidity, and bilayer organization. Positively charged liposomes increase uptake by tissues, while slightly negative ones increase circulation times due to reduced protein binding and opsonization [45,50]. Liposomes with a neutral surface charge tend to aggregate and lose their stability and colloidal behavior [51]. Biodistribution studies of nanoliposomes have shown that their half-life after intravenous injection is several hours due to hepatic uptake [52]. However, studies have shown that liposomes containing 75–100% anionic phospholipids can associate with LDL to form complexes that are then taken up by cells via LDL receptors or macrophages. This interaction highlights the potential of liposome-based targeted delivery systems for modulating LDL-related processes in various therapeutic applications [53,54].

A 2014 study [53] investigating the effects of negatively charged liposomes on lipoproteins demonstrated several beneficial outcomes. The treatment resulted in a reduction in LDL levels, total cholesterol, and ApoB, along with a notable decrease in triglycerides. At the same time, HDL levels were increased, and the liposomes showed enhanced uptake by macrophages. Moreover, the research identified atherosclerotic plaques as key target sites for anionic liposomes. These findings are particularly relevant because changes in these lipid components are directly linked to the risk of cardiovascular diseases, including atherosclerosis [53].

Building on these findings, a 2021 examination [55] in rabbits fed a high-cholesterol diet reported similar results and demonstrated significant reductions in triglycerides, total cholesterol, and LDL cholesterol levels. In addition, an increase in HDL cholesterol was observed. These findings support the potential of interventions targeting lipid profiles to manage hyperlipidemia and reduce cardiovascular risks. A 2018 study [56] conducted on a mouse model fed a high-fat diet revealed promising effects of anionic nanoliposomes in reducing the severity of atherosclerosis. The mice treated with these nanoliposomes showed decreased macrophage presence in atherosclerotic plaques. They also exhibited increased collagen deposition within the fibrous cap of plaques in the brachiocephalic artery (BCA) compared to control groups, suggesting a conceivable plaque-stabilizing effect [56]. This stabilization may have remarkable implications for reducing events related to advanced atherosclerosis. Additionally, the administration of nanoliposomes reduced the number of pro-inflammatory monocytes in both the spleen and BCA plaques of Ldlr−/− mice. It also limited hepatic steatosis and enhanced the expression of liver genes involved in lipid metabolism. Furthermore, nanoliposomes increased the aortic expression of ABCA1 and ABCG1, the key genes involved in reverse cholesterol transport (RCT). These findings highlight the potential of nanoliposomes to modulate inflammation, improve lipid homeostasis, and stabilize atherosclerotic plaques. This makes them a promising avenue for therapeutic strategies against cardiovascular disease [56].

Research suggests that negatively charged nanoliposomes hold significant capacity for reducing the risk of cardiovascular events associated with atherosclerosis and for managing dyslipidemia. By targeting key mechanisms such as LDL reduction, macrophage uptake, and plaque stabilization, these innovative nanocarriers offer an innovative strategy in pharmacology. Their ability to improve lipid profiles and influence plaque composition positions them as a hopeful development in the treatment and prevention of cardiovascular diseases.

### 5.2. Enhancing Statin Delivery via Nanoparticles

Medications used to treat hyperlipidemia are categorized into several classes, each employing distinct mechanisms to manage lipid levels. Statins are a cornerstone in the treatment of hyperlipidemia, playing a key role in lowering cholesterol levels. Their use has been linked to significant reductions in both cardiovascular-related and overall mortality rates. This impact has been observed in the prevention of atherosclerotic cardiovascular disease, benefiting patients in both primary prevention (before the onset of disease) and secondary prevention (after cardiovascular events) [57].

Statins are the primary treatment for lowering LDL-C; however, long-term adherence to this therapy is often suboptimal. Approximately 10% of the statin prescriptions are discontinued, which has been linked to a higher risk of cardiovascular events. The primary reason for discontinuation is the side effects attributed to statin. Among the various adverse effects associated with statins, muscle-related symptoms are the most frequently reported, others include hepatotoxicity, diabetes mellitus, renal impairment, and central nervous system dysfunction [58]. Although statins slightly increase the risk of mild side effects in individuals without cardiovascular disease, these effects are outweighed by their benefits in reducing major cardiovascular events. The current evidence does not strongly support the need to adjust statin type or dosage in advance to mitigate potential side effects, as limited data suggest significant variation in safety profiles across different statin regimens. This highlights the favorable balance of benefits to risks for statin use in the primary prevention of cardiovascular disease. Improving statin use in primary prevention could involve a tailored approach, adjusting the type and dose to maximize benefits while minimizing risks [59].

**Figure 1:**
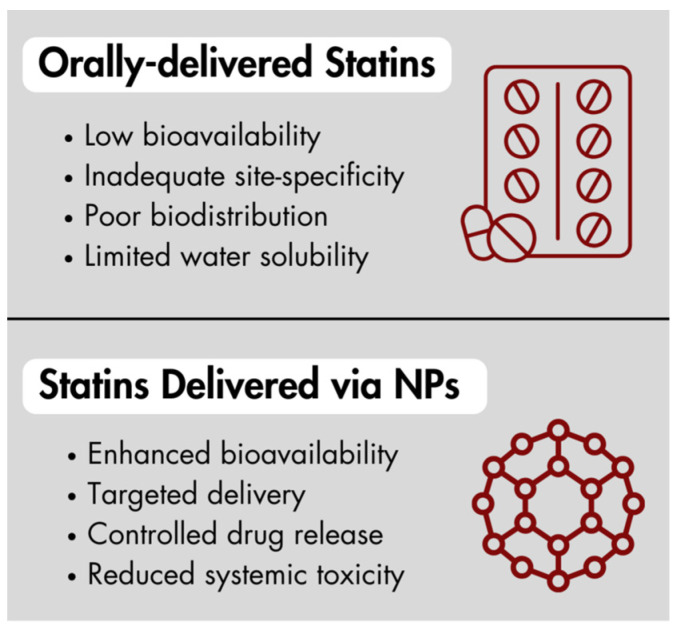
Comparison of orally delivered statins and statins delivered via NPs; NPs—nanoparticles [59].

Anti-hyperlipidemic drugs, particularly statins, face several challenges due to their pharmacokinetic and biopharmaceutical limitations. While these drugs are well absorbed, they undergo extensive hepatic first-pass metabolism, resulting in low absolute bioavailability. This metabolic process significantly reduces their systemic effectiveness. Additionally, conventional oral dosage forms are associated with several drawbacks, including poor biodistribution, low bioavailability, limited water solubility, and inadequate site-specificity. As a result, improving the delivery mechanisms of these drugs is a key area of research to enhance their clinical effectiveness and safety profile [60] (Figure 1).

As previously established, nanoparticles have the potential to address these challenges effectively. Their unique properties, such as controlled drug release, enhanced bioavailability, and targeted delivery, make them an excellent solution for overcoming the limitations of conventional drug formulations. Liposomal platforms are versatile nanocarriers used for drug delivery and molecular imaging, with ligand-functionalized liposomes specifically employed in cardiovascular imaging for targeted diagnostics [61].

In a 2021 study [62], simvastatin was encapsulated in nanoliposomes (referred to as LIPOSTAT) and tested on both 2D and 3D cell models, followed by intravenous administration. The liposomal formulation was stable, with a size of approximately 106.7 nm and a narrow size distribution. The formulation demonstrated promising therapeutic properties, including reduced inflammation and enhanced cholesterol efflux in 2D foam cells, as well as reduced inflammation in 3D spheroid models.

LIPOSTAT showed a controlled release profile, with only 10% and 20% of the drug released within the first two days, ensuring effective delivery to atherosclerotic plaques. It remained stable for at least two months and provided sustained drug release for over three weeks, enabling prolonged circulation and activity. The preliminary results highlighted its ability to regulate inflammatory and lipid accumulation pathways effectively. Specifically, LIPOSTAT significantly reduced pro-inflammatory cytokines such as IL-1α, IL-1β, and IL-18, which are key players in the early phases of inflammation. This study positions LIPOSTAT as a potential therapeutic option for targeted delivery to atherosclerotic plaques.

Another significant complication of dyslipidemia is non-alcoholic fatty liver disease (NAFLD), which can progress to fibrosis. A 2024 in vitro study [63] examined the effects of simvastatin encapsulated in nanoliposomes (SIM-LipoNPs) on fibrosis-induced liver microtissues. Simvastatin has demonstrated potential benefits in managing NAFLD, and the findings revealed that liposomal simvastatin was more effective in treating fibrosis models compared to the free drug.

The study showed that lower doses of SIM-LipoNP were as effective as higher doses of free simvastatin, likely due to the activation of the KLF2-NO signaling pathway. This suggests that liposomal formulations can overcome the challenges associated with conventional simvastatin delivery, including reducing hepatic side effects and enhancing therapeutic efficacy. The nanoliposome formulation also exhibited minimal burst release, ensuring that most of the drug payload remained intact during circulation and was efficiently delivered to the targeted liver injury site.

Despite these promising results, there remains a lack of data from in vivo studies, particularly regarding the pharmacokinetics and pharmacodynamics of SIM-LipoNP in humans. Further research is necessary to fully understand the potential of liposomal statins in treating NAFLD and to optimize their use as a targeted therapy for liver disorders.

Nanoparticles for cardiovascular diseases include gold nanoparticles, nanoliposomes, dendrimers, polymeric nanoparticles, and magnetic nanoparticles, offering unique advantages [61].

Recent studies underscore the transformative potential of nanoparticles beyond just nanoliposomes in the treatment of dyslipidemia. For instance, poly(amido)amine dendrimers (PAMAMs) were shown to significantly prolong the residence time of simvastatin and facilitate its controlled release, enhancing therapeutic outcomes [64]. Similarly, polymer vesicles loaded with pravastatin demonstrated targeted delivery and controlled release, effectively inhibiting macrophage endocytosis with reduced systemic toxicity compared to free pravastatin [65]. Additionally, poly (lactide-co-glycolic acid) (PLGA) NPs carrying atorvastatin achieved comparable efficacy to marketed formulations in hyperlipidemic rats but required a significantly lower dose, resulting in negligible myotoxicity [66].

These results emphasize the potential of nanoparticle-based systems as a game-changer for the treatment of dyslipidemia by enhancing efficacy, specificity, and safety. These innovations address many limitations of conventional therapies, offering hope for more effective and safer treatments (Figure 1). However, the lack of human trials remains a significant hurdle, requiring further research to realize their full therapeutic potential. With continued advancements, these nanocarriers could transform cardiovascular and metabolic disease management, making them a promising focus for future pharmacological development.

It is also worth highlighting the role of nanoparticles in facilitating RNA delivery to target cells and the ORION clinical trials, which evaluate the efficacy and safety of inclisiran—a novel RNA-based therapy designed to lower LDL cholesterol. Inclisiran utilizes lipid nanoparticles for efficient RNA delivery, underscoring the critical integration of nanotechnology in modern therapeutics.

RNA-delivery technology using lipid nanoparticles plays a crucial role in the practical application of RNA-based therapies. The encapsulation of RNA into LNPs prevents degradation by RNAses in the bloodstream, enabling efficient delivery to target organs. As previously discussed, LNPs’ ability to infiltrate specific hepatic cell populations contributes to the design of nanomedicines with reduced hepatotoxicity and enhanced efficacy for treating diseases originating from specific cell types [67].

Nanoparticles are essential because naked and unmodified siRNA suffers from poor stability, rapid degradation by nucleases, and potential off-target effects. Nano-delivery systems, such as LNPs, address these limitations by protecting siRNA from degradation, enhancing its stability, and improving pharmacokinetic behavior, thus facilitating its therapeutic use [68].

As previously mentioned, inclisiran, a novel siRNA drug for hypercholesterolemia, exemplifies this approach. It inhibits the production of PCSK9 in hepatocytes by silencing PCSK9 mRNA translation. This results in decreased PCSK9 synthesis, allowing more LDL receptors to clear LDL cholesterol from the bloodstream. Inclisiran received FDA approval in 2021 and EMA approval in 2020 [69], becoming the first siRNA drug approved for treating hypercholesterolemia or mixed dyslipidemia, and for patients with ASCVD or heterozygous familial hypercholesterolemia (HeFH) requiring additional LDL-C reduction [70].

The efficacy and safety of inclisiran have been evaluated in several global studies, including ORION 4, 9, 10, 11, and 18 [70]. These phase III, double-blind, randomized, placebo-controlled trials have shown that patients treated with inclisiran experienced an approximately 50% reduction in LDL cholesterol levels when administered every six months Table 2 [71,72].

In summary, the integration of nanotechnology in RNA delivery, particularly through lipid nanoparticles, has revolutionized RNA-based therapeutics, enhancing their stability, efficacy, and safety. Inclisiran represents a significant advancement in this field, offering a promising therapeutic option for managing hypercholesterolemia with demonstrated clinical benefits.

### 5.3. Polymeric Nanoparticles—Drug Delivery

Many drugs used to treat dyslipidemia, such as statins, vibrates, or niacin, may have poor bioavailability or cause side effects due to systemic exposure. Polymeric NPs have been developed to enhance the efficacy and targeting of lipid-lowering therapies. These solid particles, typically ranging from 10 to 1000 nm, are composed of macromolecular polymers, either biodegradable or non-biodegradable. Common synthetic NPs include PLGA, polyvinyl imine (PEI), and polycaprolactone (PCL) [73].

These NPs can encapsulate macromolecules, protect them from enzymatic degradation, and change the dynamic behavior and tissue distribution of the encapsulated drugs in vivo. Drugs can not only be dissolved or encapsulated in the nanoparticles but also be bound or adsorbed on the surface of the polymer nanoparticles. Moreover, they show low toxicity, good biocompatibility, and biodegradation [73].

Polymeric nanoparticles show promise for treating type 1 diabetes-related complications, including oxidative stress mediated by iron in hemoglobin, dyslipidemia, and hyperglycemia via oral delivery. They have been extensively explored as delivery vehicles for lipid-lowering drugs to treat dyslipidemia. Despite their advantages, polymeric NPs have limitations, leading to the development of lipid–polymer hybrid nanoparticles (LPHNPs). LPHNPs feature a hydrophobic polymer core to encapsulate poorly water-soluble drugs, surrounded by a hydrophilic lipid shell and an outer lipid-PEG layer. There are two techniques for synthesizing LPHNPs: the Two-Step Method and the Single-Step Approach. In the first procedure, the polymer core and lipid shell are prepared separately and mixed, whereas in the second one, lipid and polymer are directly mixed and LPHNPs are self-assembled [74].

**Figure 2:**
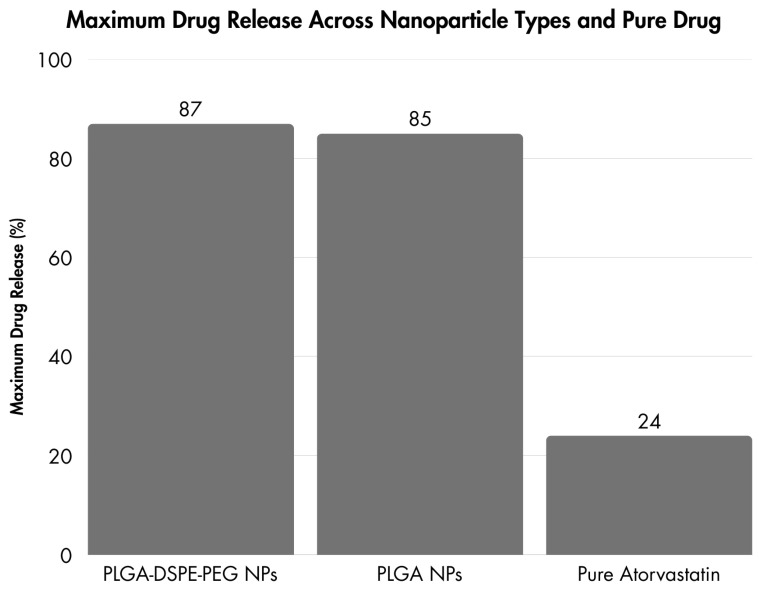
Maximum drug release across nanoparticle types and pure drug; PLGA NPs—poly lactide-co-glycolic acid nanoparticles; PLGA-DSPE-PEG NPs—polymer–lipid hybrid nanoparticles for loading atorvastatin [73].

A study conducted in 2023 demonstrated that polymeric nanoparticles and LPHNPs play a key role in the treatment of atherosclerosis, which is a complication of hyperlipidemia. Atherosclerosis is a condition when the arteries become narrowed due to the accumulation of fatty deposits, cholesterol, cellular waste products, calcium, and fibrin known as plaques located on the inner wall of the arteries. Consequently, the blood flow is reduced. This process can lead to serious cardiovascular problems such as heart attacks and strokes. The statin therapy can reduce the chances of atherosclerotic plaque formation, but it needs to be used in higher doses due to low systemic bioavailability. It can cause side effects. To overcome these challenges, nanoparticles improving systemic drug absorption and therapeutic response have been developed. Two different types of nanoparticles were prepared such as PLGA nanoparticles (polymeric) and PLGA-DSPE-PEG-nanoparticles (polymer–lipid hybrid) for loading atorvastatin. The in vitro drug release comparison was carried out for 24 h from the nanoparticles and pure drug suspension, and the obtained release profiles. The polymeric nanoparticles showed 85% drug release after 10.3 h and hybrid nanoparticles revealed 87% drug release after 8.9 h. On the contrary, the pure drug exhibited an incomplete release profile with up to 24% discharge during the entire duration of the study period (Figure 2) [73].

**Figure 3:**
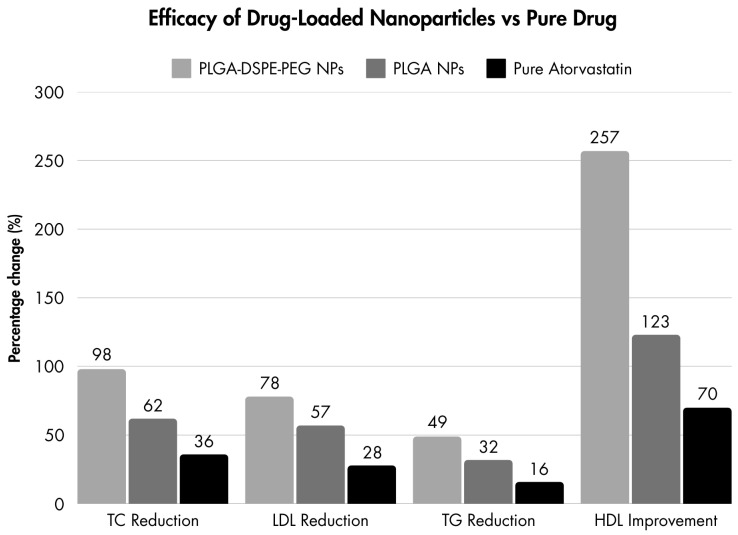
Efficacy of drug-loaded nanoparticles vs. pure drug; PLGA NPs—poly lactide-co-glycolic acid nanoparticles; PLGA-DSPE-PEG NPs—polymer–lipid hybrid nanoparticles for loading atorvastatin; TC—total cholesterol; LDL—low-density lipoprotein; TG—triglyceride; HDL—high-density lipoprotein [73].

The ability of nanoparticles to improve oral drug absorption has been widely discussed in the literature. Pharmacokinetic studies have shown that both polymeric and hybrid nanoparticles, due to their smaller particle size, facilitated better drug absorption through various mechanisms such as transcellular and paracellular transport. The drug-loaded nanoparticles demonstrated superior efficacy compared to the pure drug suspension. Specifically, the lipid–polymer hybrid nanoparticles resulted in a significantly greater reduction in the levels of TC (98%), LDL (78%), and TG (49%) compared to the control group (*p* < 0.001), while the polymeric nanoparticles led to reductions of 62%, 57%, and 32% in the TC, LDL, and TG levels, respectively (*p* < 0.001). In contrast, the animals treated with the pure drug suspension showed only a modest improvement in the TC (36%), LDL (28%), and TG (16%) levels compared to the control group (*p* < 0.05). HDL levels were also analyzed, showing improvements of 70%, 123%, and 257% after treatment with the pure drug, polymeric nanoparticles, and hybrid nanoparticles (Figure 3) [73].

The increase in drug levels in the body contributed to a stronger pharmacodynamic effect, as observed by the significant reduction in levels of hyperlipidemia markers, including TG, LDL, and TC, as well as an increase in HDL levels following nanoparticle treatment compared to the pure drug. High drug entrapment efficiency in nanoparticles is key to their improved efficacy. Both polymeric and hybrid nanoparticles showed over 70% drug entrapment, with sustained release profiles, evidenced by the time taken for 80% drug release. Polymeric nanoparticles had a more sustained release compared to lipid–polymer hybrids. The phospholipid in the hybrid nanoparticles likely boosted entrapment efficiency and drug absorption. In vivo studies also showed that the nanoparticles significantly improved systemic drug absorption through oral administration, with noticeable increases in Cmax and AUC compared to the pure drug [73].

### 5.4. Evaluating the Toxicological Impacts and Safety Strategies of Nanoparticle-Based Therapies

The potential adverse effects associated with nanoparticle-based therapies must not be overlooked. The current data on the toxicity of nanoparticles in mammalian cells and tissues underscore the need for further research to deepen our understanding of the underlying mechanisms of their toxicity. Additionally, it is imperative to develop strategies aimed at minimizing and preventing these toxic effects. Such strategies should consider both acute and chronic exposures to nanoparticles, accounting for various exposure routes and environmental contexts [74]. Below, we summarize the key side effects associated with nanoparticle-based therapies.

The toxicity of nanoparticles is influenced by their biophysical properties, including size, surface area, surface charge, and aggregation state [75]. A crucial factor in determining the efficacy or toxicity of nanoparticles is their interaction with cells. As with organ-specific toxicity, the size of nanoparticles significantly affects their cellular interactions, systemic circulation half-life, and biodistribution throughout the body [76]. The extent of endocytosis is dependent on the size of nanoparticles, with smaller nanoparticles being more readily internalized by cells, thereby increasing the likelihood of cellular toxicity. Research has also demonstrated that the shape of nanoparticles can affect their circulation time within the body, potentially delaying cellular uptake [74].

A 2018 study [77] provides a comprehensive overview of the latest findings on the side effects of nanoparticles, showing that they can accumulate in various organs, leading to different health risks. In the lungs, metal-containing nanoparticles such as cadmium-based quantum dots can induce inflammation and, in some cases, fibrosis, though recent data suggest that fibrosis may not always occur. Nanoparticles often accumulate in the liver, where they activate hepatic macrophages, contributing to liver damage. The spleen and immune system are also affected, as nanoparticles can increase cytokine levels and recruit immune cells. Small nanoparticles are typically cleared via the kidneys, but prolonged presence can lead to nephrotoxicity, including inflammation and fibrosis. Additionally, certain nanoparticles can cross the blood–brain barrier, causing hemorrhage and altering trace element levels, enzyme activity, and neurotransmitter levels after long-term exposure.

One promising approach to designing safer nanoparticles involves the development of structured nanoemulsions and solid lipid nanoparticles. These formulations utilize food-grade ingredients, generally recognized as safe (GRAS) by the FDA, such as lipids, proteins, polysaccharides, and surfactants. Research indicates that many toxic effects are associated with solid or metal-containing nanoparticles. However, further studies are necessary to assess the long-term effects and potential risks of these nanoparticles, particularly regarding their safety and side effects over extended periods [78].

### 5.5. Nanoliposomes vs. Polymeric Nanoparticles in Targeted Therapies

The targeted drug delivery system of nanoliposomes and polymeric nanoparticles has its advantages and disadvantages. Nanoliposomes may allow the successful entrapment of hydrophilic and hydrophobic drugs by their lipid bilayer composition to facilitate the required transport mechanism. However, they suffer from low criticality in terms of stability and increased clearance in circulation. On the other hand, polymeric nanoparticles, which are prepared from biodegradable polymers, showed better stability and enhanced drug release capabilities. That will lead to increasing the therapeutic effects of a certain drug. However, their practical application may be restricted by several factors such as complicated synthesis procedures and a possible cytotoxic effect [48,73,74].

## 6. Artificial Intelligence and Machine Learning in Dyslipidemia Management

With the ongoing advancements in lipid-lowering therapies, emerging technologies—such as the abovementioned nanotechnology—are reshaping the possibilities for targeted drug delivery and precision medicine. Building on these advancements, AI algorithms like ML and deep learning (DL) are probably going to revolutionize this field by enhancing the design, optimization, and application of nanomaterials [79]. The primary aim of this narrative review is to explore a novel approach to dyslipidemia management—nanotechnology. Additionally, it highlights the existing gap in the current medical approaches, specifically the potential of integrating AI with nanotechnology to enhance the dyslipidemia treatment in cardiovascular diseases.

### 6.1. Demystifying AI

Defining AI is challenging, as there is no universally accepted definition of the concept. However, a straightforward way to understand AI is as the simulation of human intelligence by systems or machines [80]. The primary functions of AI include perceiving, learning, planning, predicting, and decision making, among others. AI encompasses a wide range of research fields, such as search algorithms, knowledge graphs, expert systems, evolutionary algorithms, and advanced techniques like ML and DL. These fields contribute to AI’s ability to analyze data, solve complex problems, and adapt to new information [81].

Physicians should develop a deeper understanding of AI applications in medicine and become familiar with related concepts, as AI holds the potential to transform medical practice in unprecedented ways. Enhanced awareness and knowledge in this area will better equip healthcare professionals to harness AI-driven innovations for improved patient care and clinical outcomes [82].

Such innovation is the use of AI in pharmacogenomics, where ML and deep learning techniques are revolutionizing personalized medicine. AI enhances the prediction of drug responses, identifies genetic markers, and refines therapeutic strategies. This integration supports the development of treatment plans, minimizes adverse effects, and advances patient-specific drug therapies, contributing to more effective and individualized care [83].

### 6.2. Use of AI in Dyslipidemia Management

#### Predicting Dyslipidemia Incident

Addressing the need for more precise and personalized approaches, AI is poised to transform dyslipidemia management by improving diagnostics and enhancing treatment strategies and cardiovascular outcomes. There was a cohort study conducted in Mexico City that investigated the application of machine learning models to identify specific characteristics connected to the factors for various types of dyslipidemia [84]. It consisted of 2621 participants aged between 20 and 50 years. These patients were men and women with and without dyslipidemia. Researchers applied the Variable Importance Measures (VIMs) of random forest (RF), XGBoost, and Gradient Boosting Machine (GBM) to enable feature selection. The study revealed that the VIM algorithm of RF and GBM identified the key risk factors of dyslipidemia the most effectively (accuracy rates up to 80%). ML techniques highlighted body mass index, elevated uric acid levels, age, sleep disorders, and anxiety as the top predictors of dyslipidemia risk [84]. Presented research demonstrates ML’s ability to analyze the complex and gender-specific risk factors of dyslipidemia. Moreover, the adaptability of ML models—combined with techniques like the Synthetic Minority Over-sampling Technique for dataset resampling—supports early diagnosis and personalized treatment strategies. It can make dyslipidemia management more effective and prevent cardiovascular diseases [83].

In East Azerbaijan Province, Iran, a similar study took place; it focused on predicting dyslipidemia cases using ML algorithms [85]. The used data were obtained from the Lifestyle Promotion Project. The work results highlight the effectiveness of ML—particularly the multi-layer perception (MLP) neural network—in achieving high-reliability metrics. RF also demonstrated strong predictive capabilities, as mentioned in a previous, Mexico City study [84]. These findings emphasize the value of ML in identifying key risk factors, such as waist circumference, serum vitamin D, blood pressure, and physical activity, to enhance prediction and management strategies [85].

AI can also be used to make more accurate predictions of LDL-C levels, which is crucial for assessing atherosclerotic heart disease risk. The study, based on data from the Laboratory Information Systems of Ankara Etlik City Hospital, included 60,217 patients with lipid profiles (total cholesterol, high-density cholesterol, and triglycerides). In this research, AI models such as RF and GBM demonstrated superior accuracy compared to traditional calculation formulas, showing a stronger correlation with directly measured LDL-C values. These findings underscore AI’s potential to deliver both accurate and interpretable LDL-C predictions, enhancing clinical decision making and personalized patient care [86].

The provided studies highlight the potential of AI and ML in improving the accuracy of dyslipidemia diagnosis, risk factor identification, and LDL-C prediction. By leveraging these advanced technologies, healthcare providers can implement more precise, personalized, and effective strategies for preventing and managing cardiovascular diseases.

ML methods are still under evaluation, and their effectiveness may vary depending on the clinical context. Most clinical studies currently rely on the Friedewald formula (FF), which, despite being developed 50 years ago, remains widely used due to its simplicity, even though more accurate methods for estimating LDL cholesterol (LDL-C) are available [87].

A 2022 study compared the predictive performance of three ML models—random forests, XGBoost, and support vector regression (SVR)—against the traditional linear regression model and existing LDL-C calculation formulas in an eastern Indian population. The results showed that LDL-C predicted by the XGBoost and random forests models had a strong correlation with directly measured LDL-C (r = 0.98), outperforming the six commonly used formulas, including Friedewald, particularly across different triglyceride levels [88].

Additionally, a 2021 study on the Sampson formula found it to have the highest accuracy among various formulas, emphasizing its importance in avoiding the misclassification of hypercholesterolemia, which could delay treatment and lead to CVD progression [89]. Another 2022 study comparing the Friedewald, Sampson, and Martin–Hopkins formulas concluded that the extended Martin–Hopkins formula provided the most concordant results with direct LDL-C assays, especially in patients with low LDL-C levels or hypertriglyceridemia. However, it noted that the performance of these methods could vary with different assays and populations, highlighting the need for further validation [90].

In summary, while ML models show promise in providing more accurate LDL-C predictions, their effectiveness needs further validation across diverse clinical settings. Traditional formulas like Friedewald continue to be used but newer methods, such as the extended Martin–Hopkins and Sampson formulas, demonstrate potential for improved accuracy and require continued investigation to confirm their validity across various populations.

### 6.3. Personalized Statin Treatment Using Machine Learning

As mentioned before, AI and ML offer promising tools for personalized treatment strategies. There is research on statin therapy for individual patients that aims to enhance treatment efficacy by predicting responses and optimizing dosage based on personal risk factors. Statins are well-known and widely used medications for treating dyslipidemia. Nevertheless, they can cause side effects, such as an increased risk of diabetes mellitus, myalgia, and elevated hepatic transaminase levels [91]. This underscores the importance of personalized statin treatment.

The current guidelines provide LDL-C targets but often fail to consider individual patient responses to statins, highlighting the need for personalized therapy. In the study conducted on data sourced from patients starting statins between 2003 and 2022, a machine learning algorithm was developed using electronic health records from three tertiary hospitals (21,500 patients). The XGBoost model demonstrated the highest predictive performance, with an AUROC of 0.84 during internal validation and 0.81 during external validation, outperforming other methods like k-nearest neighbors (KNNs), support vector machine (SVM), and random forest. This model identified key factors like diabetes, baseline LDL-C, HbA1c, age, and SCORE2/SCORE2-OP as critical for predicting LDL-C target attainment. Implementing the XGB model in clinical practice could increase the likelihood of achieving LDL-C targets with initial statin prescriptions to 71–80%, offering a promising approach for personalized dyslipidemia management [92].

Another work providing a novel perspective on statin therapy explored a neural network trained to replicate National Institute for Health and Care Excellence guidelines, which was then enhanced through transfer learning using real-world outcomes from anonymized UK primary care data. The model analyzed 9675 patients receiving statin therapy and identified that for approximately 10% of the patients, smaller statin doses achieved better cholesterol reduction than higher doses recommended by guidelines. This deviation was potentially linked to improved adherence due to fewer side effects [93].

These studies highlight the potential of ML to revolutionize statin therapy by offering personalized treatment strategies that improve efficacy, optimize dosage, and enhance patient adherence through tailored care approaches.

### 6.4. Possible Synergy Between AI and Nanotechnology

Based on the developments in AI techniques discussed in the earlier parts of this review, it is crucial to consider the possibility of synergy between AI and nanotechnology since both techniques can be combined to enhance the efficiency and precision of the processes. The application of AI combined with nanotechnology may give new opportunities in various fields of medicine. It might improve the creation of advanced materials, devices, and systems. AI-driven computational tools and ML algorithms can enhance the design of nanomaterials [79].

#### 6.4.1. Drug Delivery

By enabling precision drug delivery, personalized medicine, and advanced disease detection, AI-guided nanosystems enhance therapeutic efficacy and minimize side effects through targeted delivery and real-time monitoring. AI-based nanosensors are used for early disease detection and continuous monitoring, offering real-time data. These innovations provide an idea of how much AI can be combined with nanotechnology to reform healthcare [94]. AI can predict how nanoparticles interact with biological systems, including drug release and toxicity, while reducing the need for repetitive experiments. Studies demonstrate AI’s potential to enhance drug delivery, analyze vascular systems, and address challenges like low nanoparticle delivery efficiency in cancer treatments [95].

While there is currently no accessible research explicitly combining nanotechnology and AI in the management of dyslipidemia, the potential for such integration is promising. To explore this possibility, insights from other medical fields, such as oncology, where the synergy between these technologies has been studied, may provide valuable guidance.

In original research from 2022, researchers have described the usage of NP delivery to tumors using ML and AI models [96]. This study applied multiple ML methods, including deep neural networks, RF, support vector machine, linear regression, and bagged models, to analyze data from the Nano-Tumor Database containing 376 datasets. To assess how NPs’ physicochemical characteristics, tumor models, and cancer kinds affect the effectiveness of tumor administration, a physiologically based pharmacokinetic (PBPK) model was used.

The deep neural network model outperformed the other ML methods in predicting NP delivery efficiency, achieving an R^2^ of 0.92 on the training dataset for maximum delivery efficiency and 0.70 on the test dataset. Cancer type significantly determined prediction accuracy, accounting for 19–29% of the variance.

While the model performed well on the training dataset, the R^2^ values on the test dataset were notably lower for delivery efficiency at 24 h and 168 h, indicating reduced predictive power for long-term efficiency. Additionally, the study used PBPK model data, which might not accurately represent in vivo complexity.

By offering insights into enhancing tumor delivery efficiency and comprehending its underlying challenges, this work shows how combining AI with PBPK modeling can improve the design of cancer nanomedicines [96].

The Food and Administration has been actively involved in developing NP-based drug delivery systems, with notable examples like Abraxane, an albumin-based NP formulation of paclitaxel, which has shown commercial success in breast cancer treatment. Synthetic polymers, such as poly D, L-lactic-co-glycolic acid, and lipid-based formulations like Intralipid, are commonly used due to their biocompatibility, biodegradability, and ability to improve bioavailability while reducing cytotoxicity. Recent advancements include polymeric nanoparticles for controlled release and enhanced tumor localization, as seen with Vyxeos, an FDA-approved NP-based chemotherapy for acute myeloid leukemia, which improves efficacy even at lower doses. Other innovations include Myocet, a liposomal doxorubicin formulation for breast cancer metastasis, and NBTXR3, hafnium oxide nanoparticles that enhance radiotherapy by increasing tumor cell death without harming healthy tissues. Numerous NP-based drug delivery systems are currently under clinical trials, promising significant advancements in cancer treatment upon future approvals [97].

Drawing from such examples from oncology, future research could explore AI-guided nanomedical systems for precise drug delivery and the personalized treatment of dyslipidemia. This interdisciplinary approach holds promise for improving patient outcomes by tailoring therapies to individual needs.

#### 6.4.2. NPs Design

Another important area that should be discussed is the integration of AI and nanotechnology to optimize nanoparticle design. This approach might make the applications of medicines more efficient and effective.

AI (particularly ML) has a key role in developing reproducible nanomaterials by predicting and optimizing their physical, mechanical, and chemical properties. By identifying crucial synthesis factors such as temperature, pressure, and electric current, AI tools enable the creation of tunable nanoarchitectures in dimensions like 0D, 1D, and 2D. The process begins with data cleaning and organization, followed by modeling using AI algorithms like artificial neural networks (ANNs) and deep neural networks (DNNs), allowing for reliable predictions of material properties. Although AI reduces experimental costs and time, challenges like large data requirements and computational power remain [98].

For instance, a Bayesian ML technique reduced the number of experimental runs needed to synthesize titanium nitride nanofilms to just eight. The optimized conditions predicted by the model resulted in high-quality nanofilms. The initial experimental conditions produced poor crystalline quality, but the model accurately identified accurate conditions for superior crystallinity and performance. Similarly, the synthesis of nanozeolite LTA was optimized using a complex ANN model, which identified five principal components explaining over 90% of the data variance. The model revealed relationships between key synthesis descriptors, such as temperature, pH, and pressure, and predicted the quantitative outputs of synthesis routes. This approach resulted in high-purity nanozeolite LTA, confirmed by XRD characterization. In this case, AI has not only been used to predict material properties but has also played a key role in advancing hierarchical nanostructures and self-assembling nanomaterials, showcasing its significant impact on nanotechnology [98].

The design and formulation of nanomaterials by the AI have also been successfully applied to lipid NPs, self-assembling drug–small-molecule nanoparticles, protein nanomaterials, and spherical nucleic acids. For instance, the AI-Guided Ionizable Lipid Engineering platform uses ANN to model lipid structures from the data collected by high-throughput screening large libraries of 12,000 lipid structures, identifying optimal lipid NP formulations. They were selected for their effectiveness in transfecting muscle cells, serving as a model for vaccine delivery, and macrophages, demonstrating the potential for immune cell modulation [99].

What is more, the integration of ML and high-throughput experimental data can be used to identify stable and self-assembled drug NPs. The researchers rapidly identified 100 self-assembling drug nanoparticles from 2.1 million pairings of 788 candidate drugs and 2686 approved excipients. Two nanoparticles, sorafenib–glycyrrhizin and terbinafine–taurocholic acid, were further characterized ex vivo and in vivo. This platform has the potential to accelerate the development of safer, more effective nanoformulations with high drug-loading capacities for diverse therapeutics [99].

There is a high potential of integrating AI with nanotechnology to improve nanoparticle design and synthesis, reducing costs and time. As AI continues to develop, its applications in nanotechnology are innovative across medicine, and that opens new ways in precision engineering and therapeutic advancements.

According to the insights from oncology, where AI-guided nanotechnology has been successfully used for precision drug delivery and optimizing NPs design, similar strategies could be applied to dyslipidemia. AI models could be able to design lipid-based nanoparticles for targeted drug delivery to reduce cholesterol plaques or regulate lipid metabolism, with real-time monitoring enabling personalized treatment adjustments.

ML algorithms could be able to predict nanoparticle behavior, addressing challenges like drug release efficiency and biocompatibility. This interdisciplinary approach holds significant promise for improving therapeutic outcomes and advancing innovative solutions in dyslipidemia management and surely needs a deeper focus.

### 6.5. Prospects, Difficulties, and Future Developments of AI in Dyslipidemia Management

The development of advanced technologies in AI is becoming increasingly widespread in various fields of medicine, including clinical lipidology. ML techniques, particularly ensemble methods, and neural networks are being used to predict dyslipidemia with high accuracy, precision, and sensitivity. The developed algorithms demonstrate great potential in accurately diagnosing dyslipidemia, which may lead to more effective disease management strategies. ML proves to be an invaluable tool in predicting dyslipidemia and associated disorders by leveraging patient data to improve diagnostic and treatment processes [100].

However, it should be remembered that easy access to data due to digitization and the development of applications such as ML carries the risk of disclosing sensitive health information of a patient without their consent. In this context, privacy refers to protection against attacks from competitors whose main goal is to extract sensitive information from the victim, leading to an unintended data breach [100].

Large datasets have a significant impact on the digital world, as more and more companies rely on data analysis to carry out daily operations. As a result, managing how our data is stored, updated, and shared is crucial for privacy. With the growing use of powerful internet-based data analysis tools, privacy has become an important social issue. The development of AI increases the risk of privacy violations. Advanced AI methods, such as deep learning, are ideal for analyzing large datasets and represent one of the most effective ways to process huge amounts of data in an acceptable timeframe.

Most people are unaware of the amount of data generated, analyzed, or exchanged by their devices and applications, which leads to privacy violations [100].

Despite the increase in studies applying these innovative techniques, familiarizing medical personnel with new methods and introducing new tools into clinical practice remains a challenge. The key difference is that traditional statistics rely on predefined models, whereas AI and ML are data-driven, operating without a prior understanding of the relationship between data and outcomes [101].

The ethical concerns related to the use of “black-box” medical algorithms by doctors should be further developed. It is widely acknowledged that ML algorithms carry immense potential. They offer opportunities for analyzing large datasets, recognizing patterns, and making predictions, especially in the decision-making process. AI is becoming a powerful tool, delivering promising results across various applications.

However, despite the tremendous prospects of AI revolutionizing healthcare, the functioning of some advanced ML systems remains opaque. This is due to the fact that deep learning algorithms are complex structures, whose results can be difficult to understand even for researchers, as they cannot precisely determine why the algorithm produced a specific outcome.

Such a model of operation raises concerns about values like fairness, the authority of doctors, and the privacy of patient data, as the reasons behind AI-generated decisions may also be impossible for doctors to explain. Consequently, there is a significant risk that the validity of diagnoses and recommendations for medication will be questioned by patients [102].

High-dimensional data, such as multi-omics data used in personalized health, exhibit significant inter-individual variability and are strongly influenced by environmental factors and lifestyle. Studies also indicate systematic differences in the microbiome between various ethnic groups. People from different regions of the world possess unique microbial taxa that are more abundantly represented in their bodies, likely due to both genetic and environmental differences [103].

A major ML challenge is to ensure that predictive models used in personalized diets and disease management function reliably across diverse racial, ethnic, gender, cultural, and geographic groups. The risk of algorithmic bias is particularly high in personalized health, as both the source data and individual outcomes are highly heterogeneous [104].

The creation of clear regulatory frameworks for artificial intelligence in healthcare is inevitable to ensure its implementation and deployment with consideration for safety and ethical issues. These guidelines should be flexible enough to adapt to technological advancements and emerging challenges. Key to this process will be the involvement of stakeholders who will collaboratively develop principles that account for patient safety and ethical concerns related to the development and application of AI. Among the most important regulatory issues is the standardization of performance indicators, which will enable the calculation of AI efficiency and allow the comparison of different studies and applications, contributing to their transparency. Additionally, regulatory bodies should develop clear guidelines for the safe use of AI systems, including testing protocols, validation, and ongoing oversight. Furthermore, the involvement of a broad range of stakeholders, from clinicians and patients to AI developers, in the regulatory process will ensure that various viewpoints are considered when creating standards [105].

Health disparities related to ethnicity, sex, gender identity, geographic location, and socioeconomic status still exist and are often amplified by artificial intelligence. However, these studies typically treat each factor separately—race/ethnicity, sex, gender identity, socioeconomic status, abilities, etc. What is needed now are intersectional analyses in health and medical research. Intersectionality examines how overlapping or intersecting forms of discrimination related to a patient’s social and cultural life course impact health outcomes. An iconic example of intersectional analysis in the context of facial recognition is a study that showed systems analyzing sex and race separately failed to capture the full extent of bias against black women. The sex analysis revealed that systems performed better on men’s faces than on women’s faces. The race analysis showed that systems performed better on lighter-skinned faces than on darker-skinned ones. The intersectional analysis revealed that these single axes missed the fact that systems performed much worse for black women. Further research is needed to understand how intersecting human characteristics such as sex, gender identity, race/ethnicity, socioeconomic status, and age influence health outcomes across society [106].

Currently, AI is not self-sufficient. It cannot reason in the same way that humans do. It lacks qualities such as clinical intuition and experience. Furthermore, AI does not possess developed critical thinking skills or the ability to question certain information. Instead, AI functions as a signal translator, transforming patterns from datasets. At present, AI systems are increasingly being implemented by healthcare organizations to automate time-consuming, repetitive tasks with high volumes. Additionally, significant progress has been made in applying AI to precision diagnostics (e.g., diabetic retinopathy and radiotherapy planning) [107].

The application of AI, ML, and DL in lipidology enables more accurate and clinically relevant predictions, which could significantly impact clinical practice.

Significant progress is expected in the development of powerful algorithms over the next few years, which will be efficient and capable of utilizing unstructured data as well as combining diverse data. In addition, healthcare organizations and medical practices will strive to have AI systems collaborate with technology partners in the development of new AI systems for precision therapies. However, it is crucial to understand the barriers that patients face when using lipid-lowering medications. Caution in this regard can help physicians effectively address these challenges. Moreover, in the near future, we can expect the development of improved predictive models based on lipid profiles. Additionally, innovative AI techniques could uncover previously unknown disease correlations and facilitate the implementation of precision medicine. Nonetheless, the social, methodological, and ethical complexities associated with these applications require further research and regulation.

### 6.6. Limitations and Challenges Associated with Nanoparticles and Artificial Intelligence: New Perspectives and Difficulties

Polymeric nanoparticles are a key tool in enhancing drug bioavailability and delivering them precisely to their site of action. Thanks to their versatility, polymers present a promising option capable of meeting the demands of various drug delivery systems used in specific therapies. With the increasing demand for nanoparticles, scaling up production processes becomes inevitable. This involves transitioning from small-scale production, such as in a laboratory, to larger-scale production suitable for industrial and commercial applications. The challenges associated with this process pertain to controlling nanoparticle properties (maintaining quality, uniformity, cost, and efficiency), as the synthesis techniques for PNPs at the laboratory scale often struggle with batch-to-batch variability. Scaling up production introduces numerous difficulties that need to be addressed to ensure efficiency and product quality control. This literature review focuses on several aspects of this problem [108].

Firstly, maintaining consistent properties (e.g., size, shape, charge, and polydispersity) of nanoparticles during mass production is more challenging. Ensuring that nanoparticle properties align with application requirements is crucial. During the scaling of laboratory techniques, reproducibility is key for real-world nanomaterial applications. Unfortunately, beneficial nanoparticle properties are sometimes lost. Particles may change over time or in response to environmental conditions. The temporal gap between analysis and application, as well as ensuring consistency or verification, is critical. A significant obstacle in this field is the diversity of the literature regarding research conducted and experimental data. However, there are ways to address this. Keeping records to identify sources and reduce particle variability, as well as implementing a “minimum standard of information” in nanoparticle research, including details of materials and experimental procedures, are recommended. Controlled replication studies are fundamental to avoiding excessive variations between systems and properly determining similarities and differences among compared systems.

Another important issue is nanotoxicology, which raises many concerns, particularly regarding toxicity and cellular uptake. The toxicity of nanoparticles depends, among other factors, on their chemical composition, shape, surface charge, aggregation, and solubility. After systemic exposure, they accumulate in the phagocytic system of parenchymal organs and cause DNA damage, cell cycle arrest, and ultimately cell death. Identifying the key physical or chemical characteristics of nanoparticles that contribute to their toxicity will help develop safer methods to minimize toxicity. However, this does not apply to polymeric nanoparticles, which are biocompatible, biodegradable, and non-toxic. This makes them safe for use in humans and improves drug bioavailability [108].

The next challenge in nanoparticle production is the need to consider environmental aspects, such as impact and safety. Currently, the production of various inorganic NPs with specific chemical compositions, sizes, and shapes is carried out using microbial fermentation (bacteria, yeasts, and fungi). Green nanotechnology, encompassing regulatory processes, purification, and remediation, supports environmental protection, waste reduction, and the use of safer solvents and renewable raw materials. Integrating the principles of green chemistry and engineering could offer an alternative to traditional NP synthesis methods. These methods rely on biological precursors and depend on parameters such as solvent, temperature, pressure, and pH.

Enhancing PNP production capacity is an unavoidable requirement for industry and human health. The transition from laboratory-scale to large-scale production, from batch systems to continuous systems, is becoming a reality. Several methods have been developed for producing nanoparticles with desirable characteristics on a large scale, including membrane extrusion, supercritical fluid technology, the use of microreactors in industry, or nanobiotechnology. Due to their numerous advantages, these methods offer hope for the development of various drug delivery systems and therapies. Nevertheless, the application of these methods to develop targeted and surface-functionalized nanoparticles on a large scale remains contentious. Nanomedicine represents a breakthrough in healthcare, and several clinical therapies are already available. Experiments that are thoroughly reported and analyzed offer hope that nanomedicine will soon transform many laboratory products into market-ready solutions for various therapies and clinical purposes [108].

AI algorithms are becoming a key component of healthcare, from diagnostics to population health management. Therefore, it is crucial to implement processes for mitigating algorithmic biases, which may lead to inequalities in care.

Addressing biases is not only about ensuring equal opportunities for optimal health outcomes but also promoting patient protection. Biased algorithms can result in situations where certain groups of patients do not receive adequate care, which can have serious consequences.

Medical institutions play a crucial role in addressing algorithmic biases. AI algorithms require evaluations in the specific context of their use, as they may be sensitive to differences between the data used during development and the data applied at the point of care for individual patients. Challenges such as algorithm drift or the emergence of new biases require continuous monitoring. Another issue is that some medical institutions are not sufficiently equipped to undertake the processes of evaluating and monitoring algorithmic biases, which may contribute to social inequalities. An alternative for those institutions without internal AI expertise is to seek assistance from developers. However, this raises concerns, as despite the best intentions, it may turn out that their algorithms lead to biased results without them realizing it [108].

To mitigate algorithmic biases, the best approach seems to be a shared responsibility model. In this model, all key partners, including medical institutions, AI developers, and regulatory bodies, take action to address the bias problem. Such an approach supports resource-limited institutions in adopting valuable AI algorithms into clinical care and ensures greater precision in controlling biases in medical institutions.

Another issue is the framework for AI algorithm transparency, which should align with the requirements of the ACA. There are already regulatory requirements for AI developers within the ONC and the FDA.

This will enable medical institutions to effectively assess and monitor algorithms, and the transparency process will build trust among patients and clinicians, fostering broader adoption. All transparency data should be stored in an open-access repository [109].

## 7. How AI and Nanotechnology Might Affect Healthcare?

AI offers transformative opportunities in healthcare but introduces systemic challenges and costs that require careful consideration. In the context of predictive disease models, their judicious application is vital to avoid inequitable treatment based on algorithmic predictions. As these models become integral to clinical practice, the ongoing evaluation of their performance and therapeutic impact is crucial for ensuring responsible and effective use.

To achieve fairness, training datasets must accurately represent the demographics of the target populations, integrating diverse patient data—including demographic, genetic, and environmental factors. This ensures the equitable prediction of disease progression and risk across all groups. Ethical considerations must be prioritized through informed consent, robust oversight, and comprehensive regulatory frameworks to prevent misuse or discrimination. A key advantage of AI is its potential to enhance personalized therapy, leading to reduced costs and improved patient outcomes. For example, pharmacogenomics leverages genetic information to optimize drug efficacy and minimize adverse effects, thereby advancing precision medicine [110].

Despite these benefits, implementing AI in healthcare presents significant financial and logistical challenges. By 2021, hospitals were projected to spend USD 6.6 billion annually on AI technologies, but these investments have yielded measurable benefits, such as reduced hospital readmissions, decreased emergency room visits, and improved adherence to treatment plans. For instance, Grady Hospital in Atlanta reported USD 4 million in savings over two years due to a 31% reduction in readmissions, attributed to an AI tool identifying at-risk patients [111].

However, challenges remain. Data privacy and cybersecurity risks, including unauthorized access to sensitive patient information, pose significant concerns. Ethical compliance and adherence to legal regulations are imperative to protect patient data. Additionally, AI systems developed without adequate medical expertise risk inaccuracies and misdiagnoses, compounded by a lack of transparency, errors in design, and limitations in handling unstructured data like medical imaging. The absence of standardized data further exacerbates inconsistencies across healthcare settings [112,113].

Successful AI implementation in healthcare requires addressing key issues. These include establishing ethical and secure data access processes, leveraging domain expertise to interpret data effectively, and ensuring sufficient computing power for real-time decision making, enabled by advancements in cloud computing. Rigorous research into the real-world integration of AI algorithms is essential to ensure their trustworthiness, efficacy, and seamless adoption within clinical workflows [107].

## 8. Conclusions

Dyslipidemia, characterized by abnormal lipid levels in the blood, is a significant risk factor for CVD and obesity-related complications. The abnormal lipid profile is commonly associated with obesity and closely linked to the development of atherosclerosis and thesubsequent cardiovascular events. Understanding the molecular mechanisms behind lipid metabolism and lipoprotein transport has paved the way for innovative therapeutic strategies, including the use of nanotechnology in drug delivery.

Nanotechnology, particularly through the development of nanoparticles like liposomes, holds promise in improving the delivery and efficacy of treatments for dyslipidemia. Liposomes, with their ability to encapsulate both hydrophilic and hydrophobic drugs, offer targeted drug delivery, potentially enhancing treatment outcomes while minimizing side effects. Negative-charged nanoliposomes, in particular, have shown potential in improving lipid profiles, stabilizing atherosclerotic plaques, and modulating inflammation, all of which are key factors in the management of cardiovascular diseases.

While statins remain the cornerstone of dyslipidemia treatment, their side effects, such as muscle pain, hepatotoxicity, and diabetes risk, limit their long-term adherence and effectiveness. Research indicates that combining statin therapy with innovative approaches like nanoliposome-based treatments could potentially enhance therapeutic outcomes, reduce side effects, and better manage cardiovascular risk.

Emerging evidence from clinical studies and the use of AI in predicting patient responses to treatments suggests that personalized approaches to statin therapy can optimize efficacy and minimize risks. ML models have demonstrated the ability to tailor statin doses and improve adherence, ultimately improving outcomes for dyslipidemia management.

In conclusion, while challenges remain in optimizing the current therapies and developing new treatments, advancements in nanotechnology, personalized medicine, and ML offer promising avenues to improve the management of dyslipidemia and reduce the risk of atherosclerotic cardiovascular diseases. Continued research and clinical trials will be crucial in determining the most effective strategies for integrating these innovations into routine clinical practice.

Integrating AI with nanotechnology offers groundbreaking possibilities, particularly in medicine, where precision and efficiency are critical. The success of AI-guided nanotechnology in oncology for precision drug delivery demonstrates its transformative potential. Extending similar approaches to dyslipidemia could revolutionize its treatment, enabling the design of lipid-based nanoparticles for targeted drug delivery, real-time monitoring, and personalized therapy.

By leveraging ML algorithms to predict and optimize nanoparticle behavior, we can address challenges like drug release efficiency and biocompatibility. This interdisciplinary synergy not only enhances therapeutic outcomes but also paves the way for innovative, cost-effective solutions in managing complex conditions like dyslipidemia. The potential is immense, warranting focused research and investment to unlock new frontiers in precision medicine.

Trials combining AI with nanotechnology could involve the AI-driven analysis of nanomaterials used for targeted drug delivery, optimizing dosage and delivery mechanisms for specific patient profiles, or AI could analyze real-time data from nanosensors, monitoring biomarkers in the bloodstream and enabling early disease detection and personalized interventions. However, this requires further research to ensure the effective and ethical integration of these technologies.

## Figures and Tables

**Table 1:** Comparative overview of lipid-lowering medications [26,29,34,35,42,43].

Drug/Therapy	Mechanism of Action	Half-Life	Dosing Schedule
**Statins**	Inhibition of HMG-CoA reductase	14–19 h	Once daily
**PCSK9 inhibitors**	Reduces degradation of LDL receptors	11–20 days	Every 2–4 weeks (subcutaneous)
**Inclisiran (siRNA)**	RNA interference inhibits the production of PCSK9	Very long term (months)	0–90–180 days and every 6 months thereafter (subcutaneous)
**Ezetimibe**	Inhibits the intestinal absorption of cholesterol	22 h	Once daily
**Fibrates**	Activates PPARα receptors	20 h	Once or twice daily

**Table 2:** Summary of properties of inclisiran [71,72].

INCLISIRAN	
**Structure**	siRNA conjugated to triantennary N-acetylgalactosamine carbohydrates.
**Mechanism**	Inhibits the production of PCSK9 in hepatocytes by silencing the translation of PCSK9 mRNA.
**FDA approval**	2021
**Guidelines by American College of Cardiology Expert Consensus Decision Pathway**	An option for non-statin therapy in addition to maximally tolerated statin therapy in the very-high-risk ASCVD population or those with LDL-C greater than 190 mg/dL.
**Dosage Regimen**	Initial subcutaneous dose followed by a repeat dose at 3 months and every 6 months thereafter.
**Outcomes**	Consistent LDL-C lowering in the range of 44–54%.
**Ongoing inclisiran cardiovascular outcome trials**	ORION-4, VICTORION-2 PREVENT, and VICTORION-1 PREVENT

## Data Availability

No new data were created or analyzed in this study. Data sharing is not applicable to this article.

## Abbreviations

ACA	Affordable Care Act
ACL	adenosine triphosphate-citrate lyase
ACS	acute coronary syndrome
AI	artificial intelligence
AMI	acute myocardial infarction
ANN	artificial neural network
ASCVD	atherosclerotic cardiovascular disease
BCA	brachiocephalic artery
BMI	body mass index
CAD	coronary artery disease
CK-MB	Creatine Kinase-MB
cTns	cardiac troponins I or T
CVD	cardiovascular disease
DNN	deep neural network
DHA	Docosahexaenoic acid
DL	deep learning
DM	diabetes mellitus
EPA	Eicosapentaenoic acid
FDA	Food and Drug Administration
FFA	free fatty acid
GBM	Gradient Boosting Machine
HDL	high-density lipoprotein
HDL-C	high-density lipoprotein cholesterol
H-FABP	Heart-type fatty acid-binding protein
HMG-CoA	3-hydroxy-3-methyl-glutaryl-coenzyme A reductase
HF	heart failure
IDL	VLDL remnant
KNN	k-nearest neighbor
LDL	low-density lipoprotein
LDL-C	low-density lipoprotein cholesterol
LDLR	low-density lipoprotein receptor
Lp(a)	lipoprotein (a)
LPHNPs	lipid–polymer hybrid nanoparticles
ML	machine learning
MLP	multi-layer perception
MUFA	monounsaturated fat
NAFLD	non-alcoholic fatty liver disease
NP	nanoparticle
NT-proBNP	N-terminal prohormone of brain natriuretic peptide
ONC	Office of the National Coordinator for Health Information Technology
PAMAM	poly(amido)amine dendrimer
PBPK	physiologically based pharmacokinetic
PCL	polycaprolactone
PCSK9	Proprotein convertase subtilisin/kexin type 9
PCSK9i	PCSK9 inhibitor
PEI	polyvinyl imine
PLGA	Poly(lactic-co-glycolic) acid
PRS	polygenic risk scores
PUFA	polyunsaturated fat
RCT	reverse cholesterol transport
RF	random forest
SAFA	saturated fatty acid
SIM-LipoNP	simvastatin encapsulated in nanoliposome
siRNA	small interfering RNA
SVM	support vector machine
TC	total cholesterol
TFA	trans fatty acid
TG	triglyceride
VIM	Variable Importance Measure
VLDL	very low-density lipoprotein
